# 2-Amino-3-Methylimidazo[4,5-f]quinoline Triggering Liver Damage by Inhibiting Autophagy and Inducing Endoplasmic Reticulum Stress in Zebrafish (*Danio rerio*)

**DOI:** 10.3390/toxins13110826

**Published:** 2021-11-22

**Authors:** Dan Li, Zhi Li, Tianchang Zhang, Bo Peng, Yan Zhang, Hongwen Sun, Shuo Wang

**Affiliations:** 1 Tianjin Key Laboratory of Food Science and Health, School of Medicine, Nankai University, Tianjin 300071, China; lidan@mail.nankai.edu.cn (D.L.); lizhi3204@126.com (Z.L.); tczhang@mail.nankai.edu.cn (T.Z.); iq1226jsnpb@hotmail.com (B.P.); 2 Ministry of Education Key Laboratory of Pollution Processes and Environmental Criteria, College of Environmental Science and Engineering, Nankai University, Tianjin 300071, China; sunhongwen@nankai.edu.cn

**Keywords:** HCAs, IQ, Hepatotoxicity, ERS, Autophagy, Apoptosis

## Abstract

It is important to note that 2-Amino-3-methylimidazole[4,5-f]quinoline (IQ) is one of the most common heterocyclic amines (HCAs), which is a class of mutagenic/carcinogenic harmful compounds mainly found in high-protein thermal processed foods and contaminated environments. However, the pre-carcinogenic toxicity of IQ to the liver and its mechanism are poorly understood, further research is needed. In light of this, we exposed zebrafish to IQ (0, 8, 80, and 800 ng/mL) for 35 days, followed by comprehensive experimental studies. Histopathological and ultrastructural analysis showed that hepatocytes were damaged. TUNEL results showed that IQ induced apoptosis of liver cells, the expression of apoptosis factor gene was significantly increased, and the expression of Bcl-2 protein was significantly decreased. In addition, upregulated expression of the 78-kDa glucose-regulated protein (GRP78) and C/EBP homologous protein (CHOP) and endoplasmic reticulum stress (ERS)-related factors transcription levels were elevated obviously, suggesting that IQ induced ERS. Decreased protein expression of autophagy-related 5 (Atg5)-Atg12, Beclin1, and LC3-II, increased protein expression of p62, and autophagy-related factors transcription levels were significantly decreased, suggesting that IQ inhibited autophagy. Overall, our research showed that the potential harm of IQ to the liver before the occurrence of liver cancer was related to ERS and its mediated autophagy and apoptosis pathways.

## 1. Introduction

Heterocyclic amines (HCAs), a family of mutagenic/carcinogenic compounds, are mainly generated during the pyrolysis of creatine, amino acids and proteins [1]. HCAs exist extensively in the environment, such as high-protein thermally processed foods, cigarette smoke, polluted river water, the atmosphere, soil, etc. [2]. It is important to note that 2-Amino-3-methylimidazole[4,5-f]quinoline (IQ) is one of the most studied HCAs because of its highly carcinogenic and genotoxic nature [3]. In 1993, the International Agency for Research on Cancer (IARC) classified IQ as “probably carcinogenic to humans” (group 2A carcinogens) [2]. The classification, exposure pathway, existence form, and mutagenic/carcinogenic mechanism of HCAs have been extensively studied in the early stage, but so far, the scientific research system for these issues is not complete [4,5]. Many HCAs have been proved to have prominent carcinogenic and mutagenic properties, which may bring potential harm to the human body and ecological environment; however, the pre-carcinogenic/mutagenic toxicity mechanisms of HCAs remain unclear. The previous literature has demonstrated the brain, colon and stomach toxicity of HCAs [6,7,8], and our previous study has illustrated that IQ is potentially neurotoxic to zebrafish (*Danio rerio*) [2]. However, unlike with other organs, relatively little is known about liver toxicity caused by IQ exposure, especially the potential harm to the liver and its mechanism before IQ induces liver cancer. Our recent studies have shown that IQ induces oxidative stress and inflammation leading to hepatotoxic effects in zebrafish (*Danio rerio*) through MAPK and NF-κB signaling pathways [9]. However, studies on the effects of HCAs on the liver (a pivotal organ for metabolizing toxic substances) have not been sufficient, and safety risk assessment data are still lacking. IQ toxicity and its underlying mechanisms remain elusive, as does its contribution to liver damage. Concerns about dietary and environmental exposure risks are expected to continue to increase due to the toxicity of IQ to organisms, and it is essential that we fully understand the detailed toxic mechanisms of IQ.

It has been reported that the endoplasmic reticulum (ER), the central intracellular organelle, is responsible for maintaining liver cellular functions [10]. Endoplasmic reticulum stress (ERS) refers to some pathological conditions that lead to the unfolded protein response (UPR) in the ER, which ultimately disrupts the homeostasis of the ER [11,12]. Liver cell death signals are activated during sustained or severe ERS, which has been demonstrated to associate with autophagy or apoptosis as they can be initiated by common upstream signals [11,13,14]. Autophagy normally acts as a cellular protective mechanism; nevertheless, autophagy dysfunction can lead to severe liver damage, and even cancer [15,16,17]. Hepatocyte apoptosis is considered an essential step in most forms of liver damage [18,19,20]. Additionally, oxidative stress can disrupt the internal environmental balance in the ER [19,21].

In recent years, zebrafish (*Danio rerio*) have been widely used in the study of toxicology assessment. Zebrafish and humans share highly conserved liver-associated structural and functional genes [22]. Besides, due to rapid development, primary liver morphogenesis of zebrafish is completed at 48 h post-fertilization (hpf), and the liver of the zebrafish embryo is completely capable of drug metabolism at 72 hpf [23]. Studies have shown that the results of drug hepatotoxicity assessments using zebrafish have high reproducibility with mammalian model organisms [22]. Therefore, we chose zebrafish as the model organism in this study.

In order to further understand the molecular mechanism of IQ-induced liver damage, we focused on the alterations of factors related to the ERS signaling pathway induced by IQ in toxicity evaluation. We focused on the roles of autophagy and apoptosis via ERS in liver damage not only because the crosstalk of their pathways plays a critical role in liver damage [21,22,24], but also because their relationship in the liver is still not clear. Moreover, there is little literature focused on IQ toxicity evaluation related to ERS-induced autophagy defects and apoptosis, and this study can provide evidence to reveal the relationship among them. In addition, because of IQ’s ubiquitous and potential toxicity, it is important for future dietary and ecological security studies to investigate whether it changes molecular expression patterns in the liver and leads to liver damage to understand its possible effects on human body.

As IQ exposure can cause liver damage, which is commonly associated with ER stress and defective autophagy, we hypothesized that IQ could induce decline in liver function through ER stress and inhibition of autophagy. In this study, zebrafish were exposed to four concentrations of IQ (0, 8, 80, and 800 ng/mL) for 35 days. The histopathological and ultrastructural evaluations, the hepatocyte apoptosis rate, and the abundance of genes and proteins implicated in ER stress, autophagy, and apoptosis were investigated. This study could provide supplement and support for the liver damage data of IQ and a theoretical basis for the risk assessment of IQ in diet and environment.

## 2. Results

### 2.1. IQ Induced Histological Damage in Zebrafish Livers

**Figure 1:**
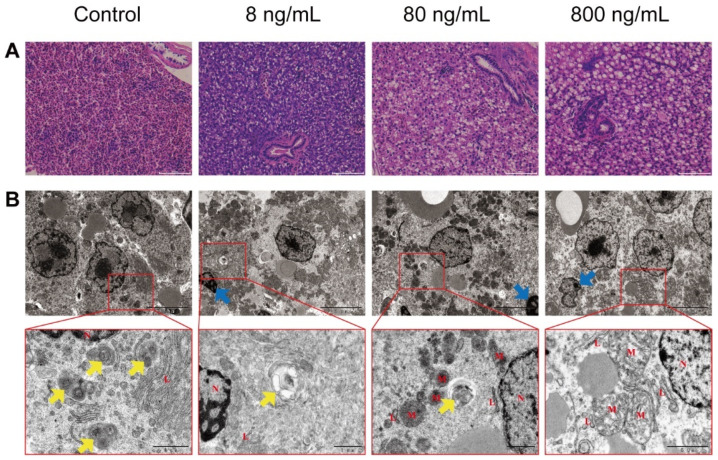
Effects of IQ on histopathology and ultrastructure in the liver of zebrafish. Representative images of each group. (**A**) Histopathological alterations in the different groups (×200, scale bar: 200 µm). (**B**) Ultrastructural observation of different groups (scale bar: 1 µm). N, cell nuclei; L, endoplasmic reticulum; M, mitochondria; yellow arrows indicated autophagic vacuoles including autophagosomes and autolysosomes; blue arrows indicated apoptosis including condensation and margination of nuclear chromatin.

The results of the H&E staining of liver tissue are shown in Figure 1A. The liver cell structure of zebrafish in the Control Group showed regular arrangement. However, in the IQ exposure groups, the liver showed noticeable pathological changes compared to the Control Group, with liver cells arranged disordered and inflammatory cell infiltration. The amount of fat vacuoles was significantly increased with increasing exposure concentration. These results suggested that IQ induced liver damage.

### 2.2. IQ Induced Ultrastructural Abnormalities in Zebrafish Livers

The results of TEM are shown in Figure 1B. Compared with the Control Group, IQ resulted in extensive abnormalities in liver cells, including vacuolation and swollen degenerated mitochondria. With increased IQ concentration, condensed and marginalized chromatin was present in the nuclei of liver cells in the IQ exposure groups, which was a typical characteristic of apoptosis. Autophagic vacuoles, including autophagosomes and autolysosomes, were observed in the cytoplasm of control hepatocytes, whereas they were less common after IQ exposure. In addition, ER damage was also observed in the same period, such as the ER was expanded, swollen, and fractured. These results suggested that IQ induced liver damage and that the damage may have been associated with ERS, autophagy, and apoptosis.

### 2.3. IQ Induced ERS in Zebrafish Livers

**Figure 2:**
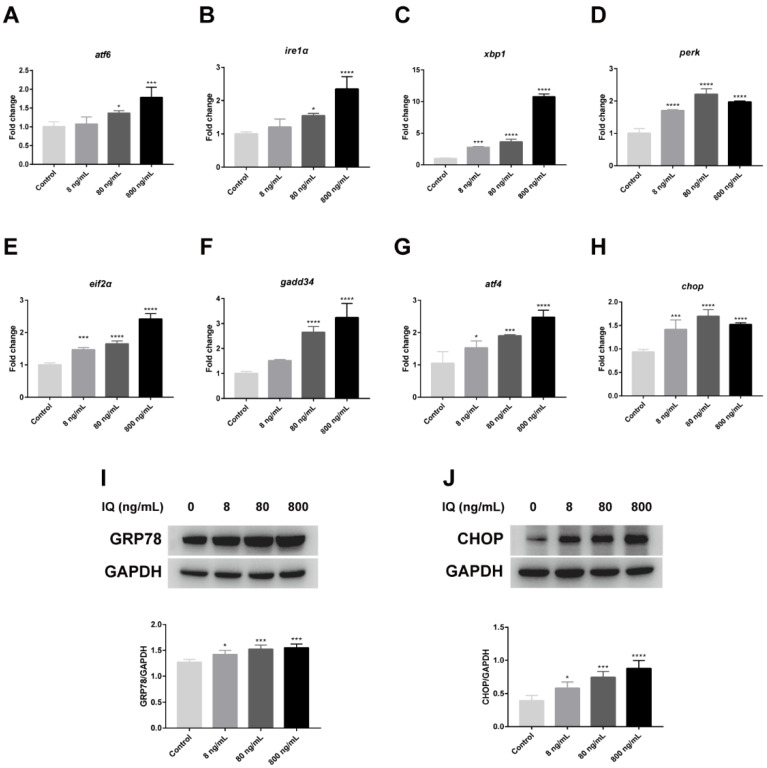
Effects of IQ on endoplasmic reticulum stress (ERS) in the liver of zebrafish. (**A**–**H**) The relative mRNA expression of *atf6*, *ire1α*, *xbp1*, *perk*, *eif2α*, *gadd34*, *atf4*, and *chop*. (**I**,**J**) Western blot analyses of GRP78 and CHOP. * *p* < 0.05, *** *p* < 0.001, **** *p* < 0.0001 (*n* = 3).

TEM revealed ERS in zebrafish livers. To confirm ERS after IQ exposure, we analyzed ERS-related genes and proteins. As presented in Figure 2A–H, the gene expression of *atf6* (*p* = 0.0002), *ire1α* (*p* < 0.0001), *xbp1* (*p* < 0.0001), *perk* (*p* < 0.0001), *eif2α* (*p* < 0.0001), *gadd34* (*p* < 0.0001), *atf4* (*p* < 0.0001), and *chop* (*p* < 0.0001) raised significantly after IQ exposure. The levels of GRP78 and CHOP, ERS marker proteins, were detected in zebrafish livers. Western blot analysis showed that GRP78 (*p* = 0.0006) and CHOP (*p* < 0.0001) were enhanced by all doses of IQ (Figure 2I,J). These results suggested that IQ induced ERS in hepatocytes.

### 2.4. IQ Inhibited Autophagic Formation in Zebrafish Livers

**Figure 3:**
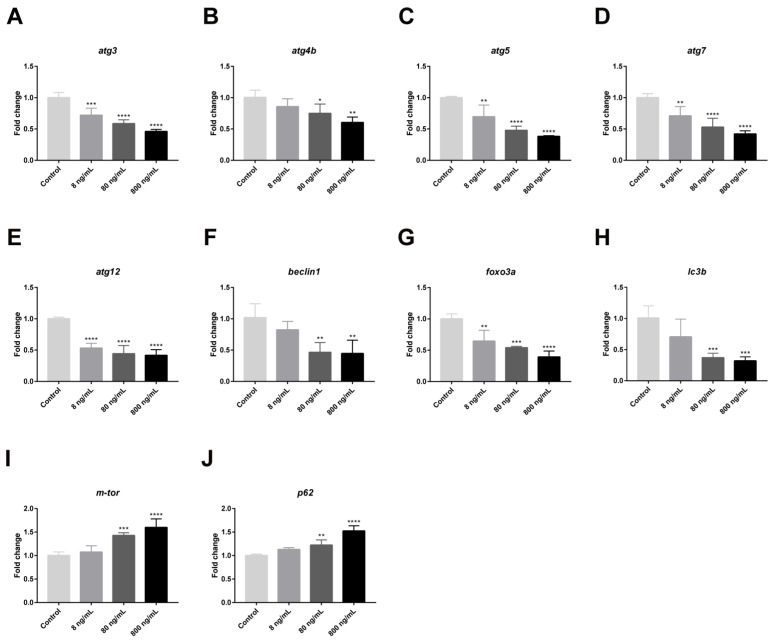
Effects of IQ on the expression of autophagy-related genes in the liver of zebrafish. (**A**–**J**) The relative mRNA expression of *atg3*, *atg4b*, *atg5*, *atg7*, *atg12*, *beclin1*, *foxo3a*, *lc3b*, *m-tor*, and *p62*. * *p* < 0.05, ** *p* < 0.01, *** *p* < 0.001, **** *p* < 0.0001 (*n* = 3).

**Figure 4:**
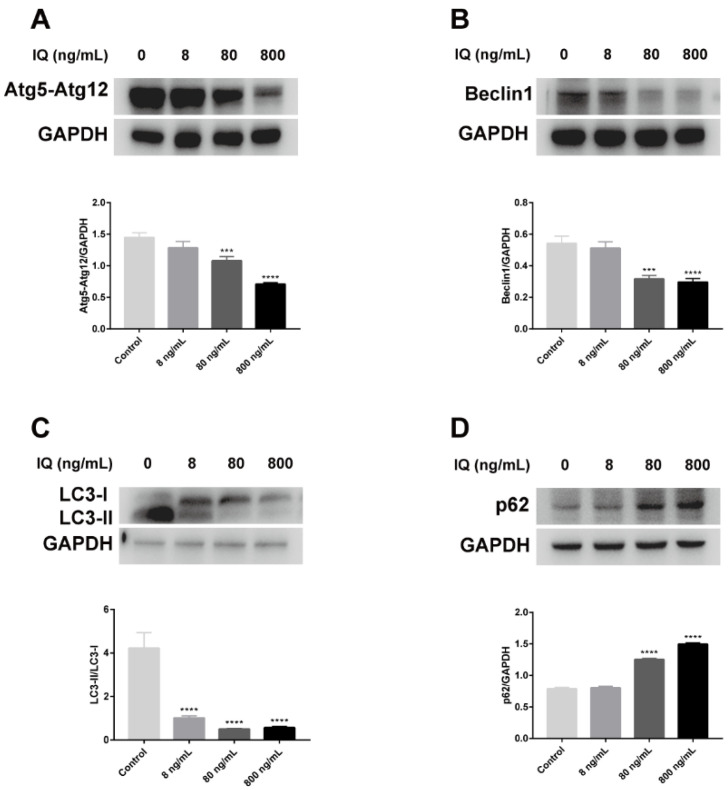
Effects of IQ on the expression of autophagy-related proteins in the liver of zebrafish. (**A**–**D**) Western blot analyses of Atg5-Atg12, Beclin1, LC3-II, and p62. *** *p* < 0.001, **** *p* < 0.0001 (*n* = 3).

TEM results showed that IQ observably inhibited autophagic formation in zebrafish livers. Autophagic vacuoles, including autophagosomes and autolysosomes, presented in hepatocytes in the Control Group, but they were rarely observed after IQ exposure (Figure 1B). To verify the alterations in autophagic response, we investigated whether the function of autophagy was impaired in zebrafish livers after IQ exposure through analyzing autophagy-related genes and proteins. As presented in Figure 3, IQ exposure significantly inhibited the gene expression of *atg3* (*p* < 0.0001), *atg4b* (*p* = 0.0037), *atg5* (*p* < 0.0001), *atg7* (*p* < 0.0001), *atg12* (*p* < 0.0001), *beclin1* (*p* = 0.0020), *foxo3a* (*p* < 0.0001), and *lc3b* (*p* = 0.0004) in a dose-related manner. The transcription of *m-tor* and *p62* significantly increased in all IQ-exposed groups (*p* < 0.0001). As shown in Figure 4, compared with the Control Groups, the protein expression levels of Atg5-Atg12, Beclin1, and LC3-II were decreased, but p62 was increased after IQ exposure (*p* < 0.0001). These results suggested that autophagic formation was inhibited after IQ exposure.

### 2.5. IQ Induced Hepatocyte Apoptosis in Zebrafish Livers

**Figure 5:**
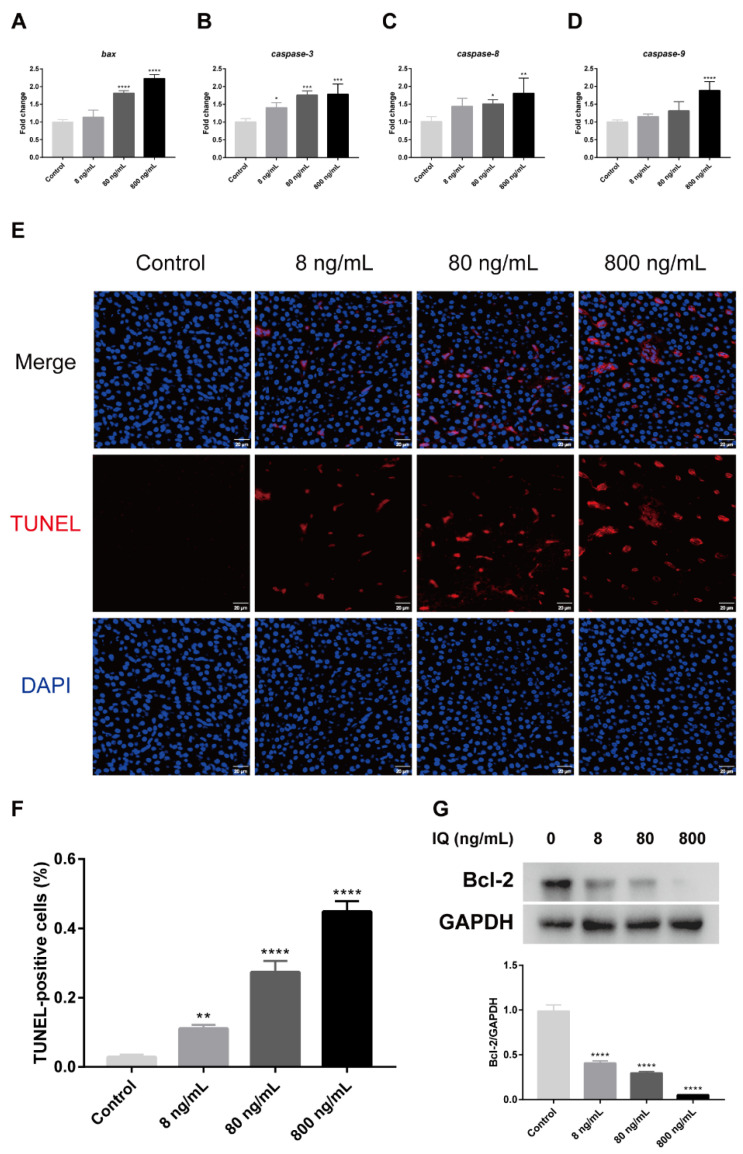
Effects of IQ on apoptosis in the liver of zebrafish. (**A**–**D**) The relative mRNA expression of *bax*, *caspase-3*, *caspase-8*, and *caspase-9*. (**E**) The effect of IQ on the apoptosis of zebrafish hepatocytes was identified using TUNEL staining (scale bar: 20 µm). (**F**) Apoptosis rates of liver cells. (**G**) Western blot analyses of Bcl-2. * *p* < 0.05, ** *p* < 0.01, *** *p* < 0.001, **** *p* < 0.0001 (*n* = 3).

To evaluate the effect of IQ on apoptotic liver cells, this study detected the levels of apoptosis-related genes and protein, as well as the percentage of apoptotic hepatocytes by TUNEL assay. As shown in Figure 5, compared with the Control Groups, the relative mRNA levels of *bax* (*p* < 0.0001), *caspase-3* (*p* = 0.0001), *caspase-8* (*p* = 0.0076), and *caspase-9* (*p* = 0.0001) were obviously increased with increasing IQ concentration in the zebrafish liver (Figure 5A–D). As shown in Figure 5E,F, the TUNEL assay showed that the hepatocyte apoptosis rate of the zebrafish liver notably increased in all IQ groups (*p* < 0.0001). In addition, we also found that the antiapoptotic protein Bcl-2 (*p* < 0.0001) were markedly decreased in the IQ-exposed groups compared to the Control Group (Figure 5G). These results indicated that IQ could induce apoptosis of hepatocytes.

## 3. Discussion

Previously available data on HCAs have mainly focused on their carcinogenic and/or mutagenic properties [1,9]. IQ, as one of the most common and mutable HCAs, has potential pre-mutagenic toxicity risks that cannot be ignored. The liver is the main organ of detoxification, and the prevention and treatment of liver damage is still a serious issue [16]. However, until now, there has been a lack of research on the liver damage effects of IQ and its mechanisms, especially the harm and potential mechanism before IQ induces liver cancer. In this study, we established an assessment model of liver damage in zebrafish with different doses of IQ exposure, focusing on the changes of factors related to the endoplasmic reticulum stress signaling pathway induced by IQ to reveal the potential molecular mechanism of IQ induced liver damage.

**Figure 6:**
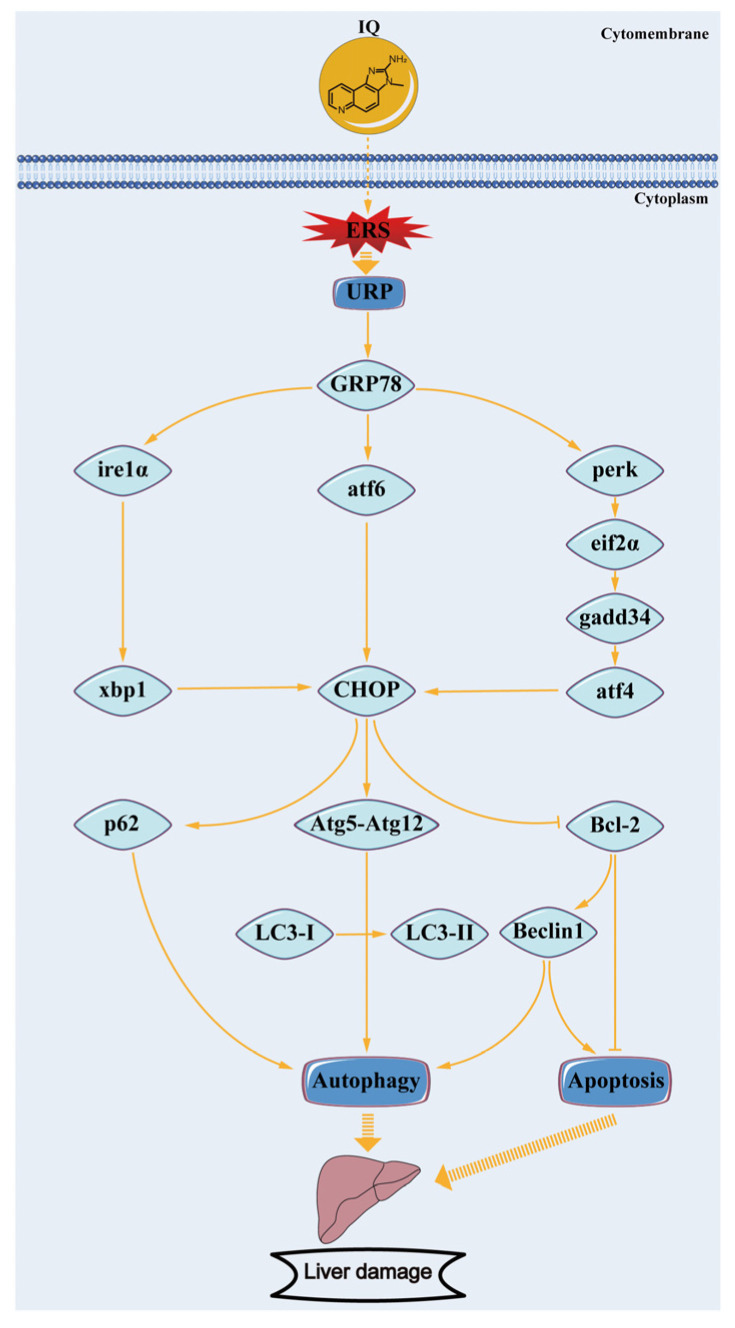
Schematic representation of endoplasmic reticulum stress (ERS), autophagy, and apoptosis in zebrafish liver induced by IQ exposure.

H&E staining results showed that IQ exposure impaired the normal structure of the liver and caused obvious pathological changes that included cells were arranged in a disordered manner, inflammatory cell infiltration and fat vacuoles were present in the liver cells, and the liver damage was more severe with increasing IQ exposure concentration. In addition, ultrastructural changes were recorded during the experiment. The results showed that the morphology of the ER and the number of autophagic vacuoles of hepatocytes in different treatment groups varied considerably. The ER is considered to be an important intracellular organelle responsible for bioactive protein synthesis, folding, posttranslational modification and delivery [25]. Accumulation of unfolded or misfolded proteins due to ER dysfunction can induce an ER stress pathway called the UPR [26]. Electron microscopy results showed that IQ exposure triggered severe ER degradation and obvious autophagy defects in hepatocytes of zebrafish, suggesting that IQ exposure induced ERS, leading to autophagy dysfunction and ultimately liver damage. ERS usually acts as a cellular defense reaction to stress in the body. It has been revealed that ERS could be triggered by the increased transcription levels of UPR-related genes (Figure 6) [27]. An appropriate ERS-induced UPR can attenuate stress and protect cells by upregulation of the protein folding mechanism [28]. However, prolonged or intense ER stress responses can lead to cell and tissue damage by regulating downstream transcription factors [22]. Plenty of researchers have reported that the development of ERS is closely related to liver diseases, such as liver fibrosis [29], hepatitis [16], and liver cancer [30]. In our study, we observed upregulation of GRP78, also known as immunoglobulin heavy chain binding protein (BIP). This molecular chaperone played an important role in UPR-induced ERS [31], indicating activation of ERS signaling in zebrafish liver. At least three transmembrane proteins mainly regulated UPR induction: inositol-requiring enzyme 1 (IRE1), activating transcription factor 6 (ATF6), and protein kinase RNA-activated-like ER kinase (PERK), and they were activated via disassociating from 78-kDa glucose-regulated protein (GRP78/BiP) when ERS occurred. After activation, the downstream related gene expression was induced respectively (Figure 6) [32]. Jia et al. reported that 3-acetyldeoxynivalenol induced liver injury through ERS, mediated by the UPR, in mice [13]. By contrast, it was reported that the ire1α inhibitor protects the liver from thioacetamide-induced liver injury [33]. In order to explore the molecular mechanism, we further examined the effects of IQ on the abundance of key genes and proteins in the UPR signaling pathways in zebrafish livers. The results showed that the IQ exposure markedly increased the gene expression of *atf6*, *ire1α*, *xbp1*, *perk*, *eif2α*, *gadd34*, *atf4*, and *chop* compared with the Control Groups, indicating that ERS participated in IQ-induced liver damage. In addition, as a pro-apoptotic transcription factor, CHOP is implicated in the ERS-dependent apoptotic response mechanism [34]. In the present study, GRP78 and CHOP expressions significantly increased after IQ exposure, revealing that the ERS-induced apoptotic pathway was involved in the hepatotoxic effect of IQ (Figure 6). Our previous study showed that livers were severely damaged because oxidative stress and inflammatory response were induced by IQ in zebrafish [9]. In addition, ER stress can trigger the oxidative stress pathways and the inflammatory pathways [12,19,21,25,33,35]. Therefore, liver damage might result from ERS, oxidative stress, and inflammation induced by IQ in zebrafish.

It is noteworthy that ERS and autophagy are highly interrelated. ER stress has long been recognized as one of the autophagy inducers [36], which can regulate autophagy through different UPR signaling pathways (Figure 6) [22]. The previous research confirmed that autophagy plays a pivotal role in various forms of liver damage [37,38,39,40]. However, there is no study about autophagy in liver after IQ exposure. Autophagy is also a cellular defense mechanism that maintains normal physiological functions of cells and tissues. However, when ERS is too long or too strong, the UPR will inhibit autophagy from inducing cell death and causing tissue damage [41]. In the autophagy process, the upstream factor Beclin1 is required for autophagy initiation and nucleation; it is also an inhibitor of tumorigenesis, and the downstream factors, such as autophagy-related gene 3 (Atg3), Atg5, and Atg7, are essential for autophagy membrane elongation and closure [42]. Several reports have shown that gene silencing of Atg3, Atg5, or Atg7 leads to impairment of autophagosome formation [42]. In addition, autophagosome completion requires two ubiquitin-like conjugation pathways: the first involves the Atg5-Atg12 conjugate, while the second involves autophagy marker Light Chain 3 (LC3) [34], which is a marker protein on the membrane of autophagic vacuoles (Figure 6). The ratios of LC3-II/LC3-I are widely used as valid markers of autophagic flux [43]. Mammalian target of rapamycin (m-TOR) is the most studied negative modulator of autophagy, and inhibition of m-TOR can activate autophagy [42]. P62, also known as sequestosome 1 (SQSTM1), is an autophagic degradation substrate, and its accumulation reflects deficiency of autophagy flux, which stimulates ERS activation and enhances ERS-induced cell damage. Interestingly, we found that IQ exposure could significantly suppress autophagy. Our results showed that IQ exposure significantly decreased the transcription levels of the autophagy-related factors *atg3*, *atg4b*, *atg5*, *atg7*, *atg12*, *beclin1*, *foxo3a*, and *lc3b*, and significantly increased the levels of *m-tor* and *p62*, in a dose-dependent manner. Correspondingly, autophagy marker proteins Atg5-Atg12, Beclin1, and LC3-II (autophagy maturation) were significantly decreased, and the protein expression of p62 was significantly increased in zebrafish liver, indicating that IQ inhibited autophagosome formation, resulting in p62 accumulation. Evidence suggested that autophagy-flux in the liver of the high-fat diet zebrafish was inhibited; it is likely that p62 carries out crucial roles on the insulin-resistance in type 2 diabetes [44]. In line with these results, autophagic vacuoles were observed in the cytoplasm of the Control Group by TEM, while almost no autophagic vacuoles were observed in the IQ exposure groups. Increasing forms of evidence has shown that hepatic injury could be ameliorated by reducing ERS and triggering autophagy [14,45]. Therefore, these findings suggest that IQ exposure can lead to inhibition of autophagy and cell damage.

In addition, accumulating evidence show that autophagy and apoptosis may share common signaling mechanisms and occur simultaneously in hepatocytes [46,47,48]. Therefore, it is particularly important to explore the relationship among ERS, autophagy, and apoptosis, which may be the potential mechanism of IQ-induced liver damage. Beclin1 deficiency has been reported to cause hepatic cell apoptosis via ERS in zebrafish larvae [49]. Meanwhile, previous work has demonstrated that ER stress is an important factor in cell death [41,50]. For example, aflatoxin B1 induced apoptosis in cells via the ERS pathway [51]. ERS can activate CHOP and induce apoptosis by attenuating the B-cell lymphoma/leukemia-2 (Bcl-2) [41]. In the present study, CHOP was significantly increased in the IQ exposure groups. Bcl-2, an antiapoptotic protein, inhibited Beclin1-dependent autophagy. Our data suggested that IQ exposure significantly increased apoptotic factors *bax*, *caspase-3*, *caspase-8*, and *caspase-9* gene expression but markedly suppressed Bcl-2 protein expression. Consistently, TUNEL analyses showed that IQ induced cell apoptosis by increasing the percentage of apoptosis. Consistent with the above results, TEM images showed condensation and margination of hepatic nuclear chromatin characterized by apoptosis. Accelerated apoptosis was a crucial toxicological mechanism of multiple toxicants exposure [24]. Furthermore, apoptosis was observed widely in liver damage [20]. Rosmarinic acid could attenuate acrylamide induced apoptosis of liver cells by inhibiting ERS [35]. Our findings indicated the crucial effect of the ERS-dependent pathway in IQ-induced hepatocyte apoptosis. The increased hepatocyte apoptosis caused by IQ exposure might be the reason for enhanced IQ hepatotoxicity in zebrafish livers. In this study, ERS played a vital role in IQ-induced inhibition of autophagy and activation of apoptosis, because crosstalk elements interact to regulate hepatocyte function under stress, suggesting the potential effect of IQ to modulate ER stress pathway, autophagic and apoptotic processes (Figure 6). These results suggested that IQ-induced hepatocyte death was multifactorial and related to autophagy and ERS-mediated responses. Our further detailed study of the mechanism of IQ hepatotoxicity is conducive to a deeper understanding of IQ toxicity, so as to lay a theoretical foundation for comprehensive assessment of food health risks and further interventions.

## 4. Conclusions

To sum up, the present study shows that IQ induced liver damage after 35 days of exposure, which is associated with ERS-mediated inhibition of autophagy and induction of apoptosis. IQ activates ERS by activating UPR, impairs liver autophagy activity and increases hepatocyte apoptosis. The harm of IQ to the liver in zebrafish before inducing liver cancer was related to oxidative stress, inflammation, and ERS-mediated autophagy defects and apoptosis. This work provides a new view for understanding and elucidating the possible causes of IQ-induced liver damage, and at the same time, have guiding significance for future research on the mechanism of other types of HCAs. In addition, higher-order species can be considered for mechanism research in the future, further, to evaluate the relationship between IQ exposure and biological mechanism.

## 5. Materials and Methods

### 5.1. Animal Treatment

Wild-type zebrafish (AB strain, and same size: 1.2–1.5 cm) aged one-month post-fertilization were cultured according to the previous method. To ensure the normal growth and nutrition of zebrafish, fresh brine shrimp were fed twice a day. Zebrafish were randomly divided into four groups and exposed to four IQ (98% purity, Toronto Research Chemicals, Ontario, Canada) concentrations: 0 (Control), 8, 80, and 800 ng/mL, respectively. Exposure concentrations were referenced from our previous studies [9]. Each group included three tanks, and each tank contained 15 fish. To maintain the water quality and IQ concentrations, all exposed solutions were replaced daily during the trial. After 35 days of IQ exposure, the zebrafish were euthanized, five fish in each treatment were selected randomly as a duplicate sample to collect liver tissues. Partial liver samples were kept at −80 °C, and the other samples were fixed, until later analysis.

### 5.2. Histology

At 35 days postexposure, zebrafish liver samples were fixed in 4% paraformaldehyde (PFA), routinely processed, sliced 5 μm thick sections after paraffin embedding, and then stained with hematoxylin and eosin (H&E). After staining, sections were observed using an electron microscope (Nikon, Tokyo, Japan) [22].

### 5.3. Transmission Electron Microscopy (TEM)

At 35 days postexposure, zebrafish livers were cut into 1-mm^3^ pieces and then were fixed with 2.5% glutaraldehyde. Next, the samples were fixed with 2% osmium tetroxide, dehydrated using ethanol and acetone. Finally, the samples were embedded in epoxy resin, ultrathin sections (Leica EM UC7, Leica, Wetzlar, Germany), and stained with uranyl acetate and lead citrate. The ultrastructure of liver tissue was then examined using a JEM1200EX transmission electron microscopy by imaging (JEM1200EX, JEOL, Tokyo, Japan) [52].

### 5.4. Quantitative Real-Time Polymerase Chain Reaction (qPCR)

Total RNA of zebrafish liver was extracted using Trizol (Thermo Fisher Scientific, Waltham, MA, USA). The gene transcription levels were determined by qPCR, as previously described. β-Actin was used as an internal reference. The relative expression levels of the target genes were analyzed with 2^−ΔΔCT^. Primer sequences are shown in Table S1.

### 5.5. Western Blot

The relative protein expression levels of Bcl-2, 78-kDa glucose-regulated protein (GRP78), C/EBP homologous protein (CHOP), autophagy-related 5-autophagy related 12 (Atg5-Atg12), Beclin1, LC3, p62, and GAPDH in the liver were measured by using Western blotting. PVDF membranes (Millipore, Billerica, MA, USA) with protein bands were obtained as previously described [9]. Membranes were blocked for 1 h using 5% BSA in TBS containing 0.5% Tween-20 (TBST) and then incubated with primary antibodies against Bcl-2 (Affinity Biosciences, Cincinnati, OH, USA), GRP78 (ProteinTech Group, Chicago, IL, USA), CHOP (ProteinTech Group, Chicago, IL, USA), ATG5 (Novus Biologicals, Littleton, CO, USA), Beclin1 (ProteinTech Group, Chicago, IL, USA), LC3 (ProteinTech Group, Chicago, IL, USA), p62 (ProteinTech Group, Chicago, IL, USA), and GAPDH (Cell Signaling Technology, Danvers, MA, USA) overnight at 4 °C. The next day, after washing with TBST three times, the membranes were incubated with secondary antibody (ProteinTech Group, Chicago, IL, USA) for 1 h followed by three additional washes with TBST at room temperature. Finally, these protein bands were stained and quantified according to a protocol previously described.

### 5.6. Terminal Deoxynucleotidyl Transferase-Mediated dUTP Nick End Labeling (TUNEL) Assay

At 35 days postexposure, zebrafish liver samples were fixed in 4% PFA, dehydrated, sliced 8 μm thick sections after optimal cutting temperature compound (OCT) (Sakura Finetek, Torrance, CA, USA) embedding. The apoptotic cells’ number was analyzed using a TUNEL Apoptosis Assay Kit (Solarbio, Beijing, China). Briefly, after washing with PBS, the sections were incubated with TUNEL working solution for 60 min at 37 °C in a dark and humidified atmosphere, and the nuclei were counterstained with DAPI. The results were observed and photographed under a confocal laser scanning microscope (FV1000, Olympus, Tokyo, Japan). Images were processed and analyzed by ImageJ software.

### 5.7. Statistical Analysis

Statistical analysis was performed with GraphPad Prism 7. Data were expressed as the means ± standard deviations (SDs). Statistical significance was evaluated by one-way ANOVA followed by Tukey’s multiple comparison test. Each experiment was performed at least three times. A *p*-value < 0.05 was considered to be significant.

## Data Availability

Data are available upon request; please contact the contributing authors.

## Supplementary Materials

The following are available online at https://www.mdpi.com/article/10.3390/toxins13110826/s1, Table S1: primer sequences for qPCR used in this study.
