# Supplementary material for: 2-Amino-3-Methylimidazo[4,5-f]quinoline Triggering Liver Damage by Inhibiting Autophagy and Inducing Endoplasmic Reticulum Stress in Zebrafish (Danio rerio)
Source: Toxins (Basel). 2021 Nov 22;13(11):826. doi: 10.3390/toxins13110826 (PMC8620671; doi:10.3390/toxins13110826)
Supplement: Supplementary file 1 [file toxins-13-00826-s001.zip › toxins-1454007-supplementary.pdf]

toxins

MDPI

Supplementary Information

2-Amino-3-methylimidazo[4,5-f]quinoline Triggering Liver Damage by Inhibiting Autophagy and Inducing Endoplasmic Reticulum Stress in Zebrafish (*Danio rerio*)

Dan Li [1], Zhi Li [1], Tianchang Zhang [1], Bo Peng [1], Yan Zhang [1,*], Hongwen Sun [2] and Shuo Wang [1,*]

[1] Tianjin Key Laboratory of Food Science and Health, School of Medicine, Nankai University, Tianjin 300071, China; lidan@mail.nankai.edu.cn (D.L.); lizhi3204@126.com (Z.L.); tczhang@mail.nankai.edu.cn (T.Z.); iq1226jsnpb@hotmail.com (B.P.)
[2] Ministry of Education Key Laboratory of Pollution Processes and Environmental Criteria, College of Environmental Science and Engineering, Nankai University, Tianjin 300071, China; sunhongwen@nankai.edu.cn
* Correspondence: yzhang@nankai.edu.cn (Y.Z.); wangshuo@nankai.edu.cn (S.W.)

Table S1. Primer sequences for qPCR used in this study.

Symbol	Forward Primer (5′-3′)	Reverse Primer (5′-3′)
bax	GGCTATTTCAACCAGGGTTCC	TGCGAATCACCAATGCTGT
caspase-3	CCGCTGCCCATCACTA	ATCCTTTCACGACCATCT
caspase-8	CCAGACAATCTGGATGAACTTTAC	TGCAAACTGCTTTATCTCATCT
caspase-9	CTGAGGCAAGCCATAATCG	AGAGGACATGGGAATAGCGT
atf6	CTGTGGTGAAACCTCCACCT	CATGGTGACCACAGGAGATG
ire1α	TGACGTGGTGGAAGTTGGTA	ACGGATCACATTGGGATGTT
xbp1	GGGTTGGATACCTTGGAAA	AGGGCCAGGGCTGTGAGTA
perk	TGGGCTCTGAAGAGTTCGAT	TGTGAGCCTTCTCCGTCTTT
eif2α	CATTGAGGTGGCGTGTTATG	TCGATCCGCTCTTTAATTGC
gadd34	TTCACCATCTCCACACCTCA	CTGCCACAGCTTCATTTTGA
atf4	TTAGCGATTGCTCCGATAGC	GCTGCGGTTTTATTCTGCTC
chop	AAGGAAAGTGCAGGAGCTGA	TCACGCTCTCCACAAGAAGA
atg3	ACCGTCACCATTGAGAATC	AGTTCACCTCCTCCTTCC
atg4b	ACACCACTCAGCCAGCAG	AAGCCAGCAGCAATAGAAGG
atg5	CAACTGTGGATGGGTCTG	GAGCGTCTGGATGAATGG
beclin1	GCCATTGTATTGTTCAGGTG	CAGAAGCCAGTGTCATCC
atg7	AGAGTCCAGTCCGATGTC	GAAGTAACAGCCGAGACG
atg12	CCAGTTCATCTCACGCTTCCTC	TGCCGTCACTTCCGAAACAC
p62	TTTGGCTCTTGTGAAGGATGAC	GAGGGCTAAAGTGAGGTGTAGTGA
foxo3a	GAGTCCAGTAGTCGCAGTG	GCAGTTCATCCGCCATCC
m-tor	ATACGCATCCAGTCCATTG	TCATTAGCCAGTAGAGTGTTC
lc3b	CCTCCAACTCAACTCCAACC	GCCGTCTTCGTCTCTTTCC
β-actin	AGGTCATCACCATTGGCAAT	GATGTCCACGTCGCACTTCAT
